# Sustaining Antimicrobial Stewardship in a High–Antibiotic Resistance Setting

**DOI:** 10.1001/jamanetworkopen.2022.10180

**Published:** 2022-05-03

**Authors:** Tat Ming Ng, Shi Thong Heng, Boon Hou Chua, Li Wei Ang, Sock Hoon Tan, Hui Lin Tay, Min Yi Yap, Jason Quek, Christine B. Teng, Barnaby E. Young, Ray Lin, Brenda Ang, Tau Hong Lee, David C. Lye

**Affiliations:** 1 Department of Pharmacy, Tan Tock Seng Hospital, Singapore, Singapore; 2 National Public Health and Epidemiology Unit, National Centre for Infectious Diseases, Singapore, Singapore; 3 Department of Pharmacy, Faculty of Science, National University of Singapore, Singapore, Singapore; 4 Department of Infectious Diseases, National Centre for Infectious Diseases, Singapore, Singapore; 5 Department of Infectious Diseases, Tan Tock Seng Hospital, Singapore, Singapore; 6 Lee Kong Chian School of Medicine, Nanyang Technological University, Singapore, Singapore; 7 Yong Loo Lin School of Medicine, National University of Singapore, Singapore, Singapore

## Abstract

**Importance:**

There is a lack of studies comparing the intended and unintended consequences of prospective review and feedback (PRF) with computerized decision support systems (CDSS), especially in the longer term in antimicrobial stewardship.

**Objective:**

To examine the outcomes associated with the sequential implementation of PRF and CDSS and changes to these interventions with long-term use of antibiotics for and incidence of multidrug resistant organisms (MDROs) and other unintended outcomes.

**Design, Setting, and Participants:**

This cohort study used an interrupted time series with segmented regression analysis of data from January 2007 to December 2018. Data were extracted from the electronic medical records of patients admitted at a large university teaching hospital with high rates of antibiotic resistance in Singapore. Data were analyzed from June 2019 to June 2020.

**Exposures:**

PRF of piperacillin-tazobactam and carbapenems (intervention 1, April 2009), with the addition of hospital-wide CDSS (intervention 2, April 2011), and lifting of CDSS for half of the hospital wards for 6 months (intervention 3, March 2017).

**Main Outcomes and Measures:**

Monthly antimicrobial use was measured in defined daily doses (DDDs) per 1000 patient-days. The monthly incidence of MDROs was calculated as number of clinical isolates detected per 1000 inpatient-days over a 6-month period. Unintended outcomes examined included in-hospital mortality and age-adjusted length of stay (LOS).

**Results:**

The number of inpatients increased from 56 263 in 2007 to 63 572 in 2018. During the same period, the mean monthly patient days increased from 33 929 in 2007 to 45 603 in 2018, and the proportion of patients older than 65 years increased from 45.5% in 2007 to 56.6% in 2018. After intervention 1, there were 0.33 (95% CI, 0.18 to 0.48) more DDDs per 1000 patient-days per month of piperacillin-tazobactam and carbapenems and −11.05 (95% CI, −15.55 to −6.55) fewer DDDs per 1000 patient-days per month for other broad-spectrum antibiotics. After intervention 2, there were −0.22 (95% CI, −0.33 to −0.10) fewer DDDs per 1000 patient-days per month of piperacillin-tazobactam and carbapenems and −2.10 (95% CI, −3.13 to −1.07) fewer DDDs per 1000 patient-days per month for other broad-spectrum antibiotics. After intervention 3, use of piperacillin-tazobactam and carbapenem increased by 0.28 (95% CI, 0.02 to 0.55) DDDs per 1000 patient-days per month. After intervention 2, incidence of *Clostridioides difficile* decreased (estimate, −0.02 [95% CI, −0.03 to −0.01] cases per 1000 patient-days per month).

**Conclusions and Relevance:**

In this cohort study, concurrent PRF and CDSS were associated with limiting the use of piperacillin-tazobactam and carbapenems while reducing use of other antibiotics.

## Key Points

**Question:**

Can the outcomes associated with an antimicrobial stewardship program be sustained in the long term?

**Findings:**

In this cohort study, after implementation of prospective review and feedback, the use trends increased for piperacillin-tazobactam and carbapenems and declined for other broad-spectrum antibiotics. After the addition of a computerized decision support system, the use trends decreased for piperacillin-tazobactam and carbapenems and for other broad-spectrum antibiotics.

**Meaning:**

These findings suggest that concurrent use of prospective review and feedback and a computerized decision support system may help to sustain the outcomes associated with antimicrobial stewardship programs.

## Teaser

This cohort study examines associations of sequential implementation of antimicrobial stewardship initiatives with trends in antibiotic use and incidence of multidrug resistant organisms.

## Introduction

Antimicrobial stewardship has become an important strategy to tackle antimicrobial resistance. In 2017, the World Health Organization (WHO) categorized antibiotics into Access, Watch, and Reserve categories. Seven classes of antimicrobials, fluoroquinolones, third-generation cephalosporins, glycopeptides, antipseudomonal penicillins with β-lactamase inhibitors, and carbapenems, were on the Watch list.^1^ These are commonly used in hospitals and thus have been targeted by antimicrobial stewardship teams for interventions.^2,3,4,5^

The effectiveness of antimicrobial stewardship activities, such as prospective review and feedback (PRF) and computerized decision support systems (CDSS), in hospitals has been demonstrated.^6,7^ However, there is a lack of studies comparing PRF with CDSS. In addition, there are calls for adopting robust study designs using interrupted time series (ITS) analysis to evaluate intended and unintended consequences.^8^

Two main interventions, PRF and CDSS, were implemented sequentially at a university teaching hospital in Singapore, where up to 37.7% of all *Enterobacterales* were not susceptible to third-generation cephalosporins and 7.0% were not susceptible to carbapenems.^9^ We aimed to examine the associations of the sequential implementation and changes to these interventions with the long-term use of antibiotics with high use, incidence of multidrug-resistant organisms (MDROs), and other unintended outcomes.

## Methods

This cohort study was approved by the Domain Specific Review Board of the National Healthcare Group, Singapore, which waived the requirement for informed consent because the study was based on aggregated nonidentifiable data. This study was reported following the Strengthening the Reporting of Observational Studies in Epidemiology (STROBE) reporting guideline for cohort studies.

### Study Design, Setting, and Interventions

We conducted an interrupted time series with segmented regression analysis of antibiotic use, MDRO incidence, and other unintended outcomes at Tan Tock Seng Hospital (TTSH), Singapore, a university teaching hospital, over 11 years, from January 2007 to December 2018. All operational wards were included. In 2007, antimicrobial stewardship interventions included antibiotic restrictions for vancomycin, linezolid, carbapenems, and polymyxin B. In 2009, a multidisciplinary antimicrobial stewardship team, including infectious disease physicians and antimicrobial stewardship–trained pharmacists, was established. The hospital implemented empirical antibiotic guidelines in January 2009, and daily real-time PRF of piperacillin-tazobactam and carbapenems was conducted on weekdays starting in April 2009 (intervention 1). Antibiotic restrictions were thereafter limited to only linezolid and polymyxin B. During the PRF, the antimicrobial stewardship team conducted audits and provided recommendations concurrently to the prescribers, who could accept or reject them. A CDSS that covered 52 infective syndromes and followed hospital empirical antibiotic guidelines was introduced in April 2011 (intervention 2). Details of the CDSS have been described elsewhere.^10,11^ Briefly, the CDSS is accessed via the electronic medication record; prescribers specify the infection they are treating and enter selected patient parameters in the system and receive a set of recommendations based on empirical hospital guidelines. Prescribers have the autonomy to override the recommendations. The guidelines do not recommend carbapenems and antipseudomonal antibiotics for community-acquired infections. Guided by hospital antibiogram, the guidelines discourage the use of ceftriaxone, ciprofloxacin, and levofloxacin, when possible. Culture-directed deescalation recommendations are also available. CDSS use was compulsory for piperacillin-tazobactam and carbapenems and voluntary for other antibiotics. This CDSS was available at all wards in the hospital. In March 2017, a 6-month cluster-randomized clinical trial with a crossover design was conducted to compare the effects between compulsory and voluntary use of CDSS for piperacillin-tazobactam and carbapenem prescriptions in 32 acute wards in the main building of the hospital.^10^ In the first month, compulsory CDSS was instituted for half of the 32 wards. In the third month, wards assigned to compulsory CDSS use were switched to voluntary CDSS use (intervention 3). PRF was continued for all wards throughout the 6 months. A total of 19 acute care wards (13 acute care wards outside of the main hospital building, 4 intensive care units, and 2 high-dependency units) and 12 subacute care wards were excluded. However, as the 32 acute care wards in the main hospital building contributed two-thirds of total patient days, it was considered a significant intervention. A crossover design was adopted to adjust for bias and contamination between clusters owing to Hawthorne and spillover effects because of physicians’ rotation and patient transfers.^10^ In both study groups, more than 80% of patients had their physicians accepting CDSS and PRF recommendations. However, patients in the voluntary CDSS wards received significantly fewer CDSS recommendations (132 recommendations for 641 prescriptions [21%]) compared with the compulsory CDSS arm (612 recommendations for 616 prescriptions [99%]).^10^

### Data Collection and Outcomes

Data on antibiotic use were extracted from the pharmacy dispensing system, and data on incidence of MDROs in TTSH were collected from the hospital electronic medical record system. We included piperacillin-tazobactam and carbapenems targeted by PRF and CDSS. In addition, antibiotics that were considered broad-spectrum and had high use were included, defined as levofloxacin, ciprofloxacin, ceftriaxone, ceftazidime, cefepime, vancomycin, and intravenous amoxicillin-clavulanate. Monthly antimicrobial use was measured in defined daily doses (DDDs) per 1000 patient-days to adjust for fluctuations in patient load. For MDROs, we included the incidence of third-generation cephalosporin–resistant (3GCR) *Klebsiella pneumoniae* and *Escherichia coli*, *Clostridioides difficile*, carbapenem-resistant *Pseudomonas aeruginosa* and carbapenem-resistant *Acinetobacter baumannii*. The monthly incidence was calculated as number of clinical isolates per 1000 inpatient-days. Only the first of repeated isolates (identical species and identical antimicrobial susceptibility) from the same patient was counted during a 6-month period. Unintended outcomes were examined in terms of in-hospital mortality and age-adjusted length of stay (LOS). These outcome data were obtained from the hospital quality management information system. In-hospital mortality was defined as number of deaths per 100 inpatient discharges (including death discharges). Age-adjusted mean LOS was calculated by dividing total patient-days in a month with number of inpatient discharges in the same month, adjusted by age, using year 2007 as the reference year. Since there was a substantial expansion of subacute care wards in the hospital over the years, we reexamined the time-series again after excluding subacute care wards.

### Statistical Analysis

Sample size calculation was not conducted, as data from all patients at the hospital were included. A segmented regression analysis of interrupted time series was used to examine changes in the level and trend. Separate intercepts and slopes were estimated in each preintervention and postintervention segment of the period.^12^ We applied ordinary least squares regression with Newey-West SEs to account for an error structure that was assumed to be heteroskedastic and autocorrelated at lag 2 for piperacillin-tazobactam and carbapenems and at lag 3 for other broad-spectrum antibiotics. The autocorrelation of each model was determined using Cumby-Huizinga general test and visual inspection of autocorrelation and partial autocorrelation plots. No apparent seasonality was detected using periodogram, cycle plot, and visual inspection of autocorrelation and partial autocorrelation plots. Nonstationarity was not detected in all the time series using Dickey-Fuller test.

We examined the lead-lag association between antimicrobial resistance and antibiotic use. To identify meaningful associations between 2 time series, each time series was prewhitened to remove autocorrelation and temporal patterns. An autoregressive integrated moving average CC model was fitted to an antibiotic use time series, which was filtered to obtain white noise residuals.^13,14^ The MDRO incidence time series was also filtered with the same model. The cross-correlation function was computed between the filtered time series for each antibiotic use and MDRO incidence at lags of up to 12 months. For cross-correlations that were statistically significant, we noted the lag in the cross-correlation function corresponding to the maximum correlation coefficient (*r*), to determine the lead time of antibiotic use relative to 3GCR *K pneumoniae* and *E coli*, *C difficile*, carbapenem-resistant *P aeruginosa*, and carbapenem-resistant *A baumannii*.^15^

All *P* values were 2-sided, and statistical significance was set at *P* < .05. Segmented regression analyses were performed using Stata Standard Edition version 16 (StataCorp) and correlation analyses were performed using R version 3.5.0 (R Project for Statistical Computing). Data were analyzed from June 2019 to June 2020.

## Results

The annual number of inpatients increased from 56 263 in 2007 to 63 572 in 2018. During the same period, the proportion of inpatients aged older than 65 years increased significantly, from 45.5% in 2007 to 56.6% in 2018 (*P* < .001). The mean (range) monthly number of operational acute care wards was 42 (40-44) wards in 2007 and 53 (53-56) wards in 2018, and operational subacute wards increased from 1 in 2007 to 10 in 2018. The mean (range) monthly patient-days also increased from 33 929 (29 723-35 028) patient-days per month in 2007 to 45 603 (43 101-48 740) patient-days per month in 2018.

### Antibiotic Use

**Figure 1.**
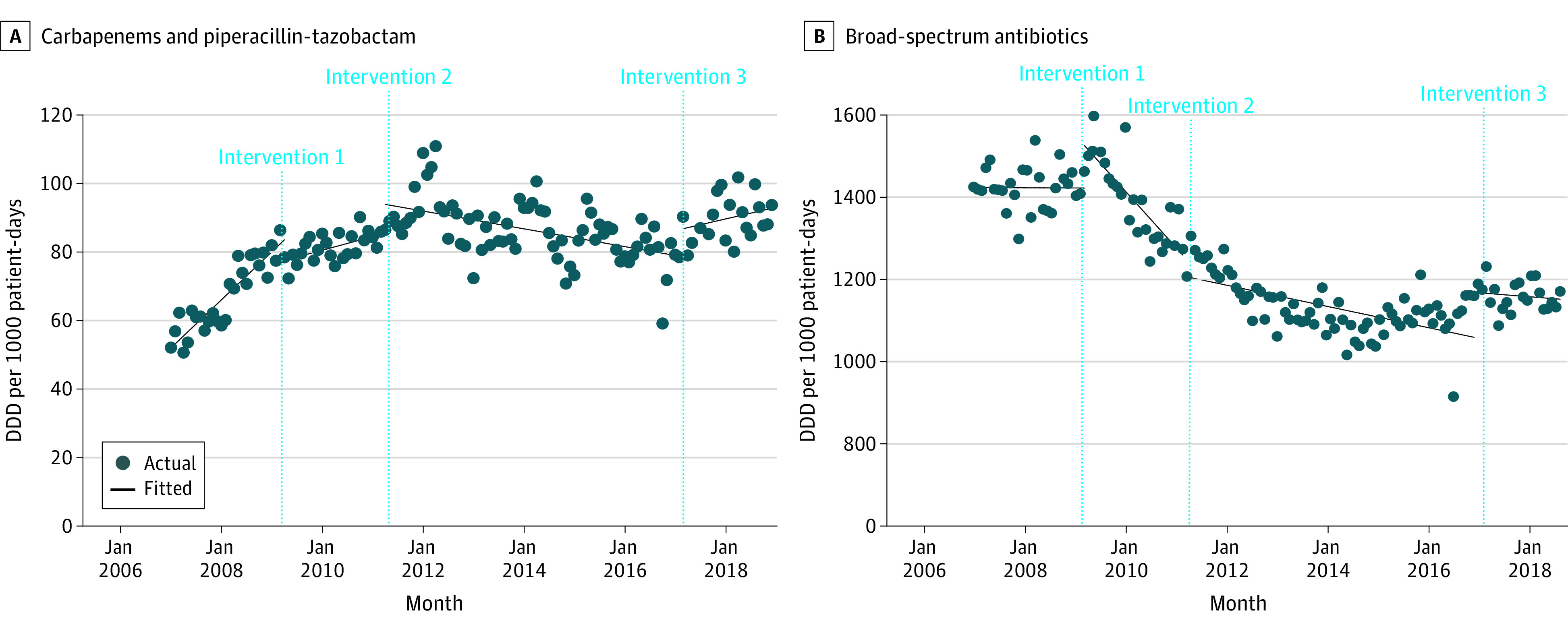
Associations of Antimicrobial Stewardship Interventions With Antibiotic Use Over Time Intervention 1 began in April 2009 and included introduction of empirical antibiotic guidelines and prospective review and feedback. Intervention 2 began in April 2011 and included compulsory use of computerized decision support systems in addition to the voluntary access to the computerized decision support systems for antibiotic recommendations. Intervention 3 began in March 2017 and included lifting of the compulsory use of computerized decision support systems for piperacillin-tazobactam and carbapenems for half of the wards in the hospital. Compulsory use was reinstated from September 2017. DDD indicates defined daily dose.

In January 2007, the level of piperacillin-tazobactam and carbapenems was 52.19 (95% CI, 49.07 to 55.3) DDDs per 1000 patient-days, and use increased at a mean monthly trend of 1.17 (95% CI, 0.99 to 1.35) more DDDs per 1000 patient-days. When empirical antibiotic guidelines and PRF were implemented in April 2009 (intervention 1), use of piperacillin-tazobactam and carbapenems increased at a slower monthly trend of 0.33 (95% CI, 0.18 to 0.48) more DDDs per 1000 patient-days. When compulsory CDSS for piperacillin-tazobactam and carbapenem prescription and voluntary use of CDSS for other antibiotics were introduced in April 2011 (intervention 2), use decreased at a trend of −0.22 (95% CI, −0.33 to −0.10) fewer DDDs per 1000 patient-days per month for piperacillin-tazobactam and carbapenems. However, there was a significant increase of 0.28 (95% CI, 0.02 to 0.55) more DDDs per 1000 patient-days per month when compulsory use of CDSS for piperacillin-tazobactam and carbapenems was lifted in March 2017 for half of the acute wards (intervention 3). All 3 interventions were associated with significant change in use level in the month of implementation and trend change (Table 1; Figure 1A).

**Table 1.** Interrupted Time Series Analysis of the Association of Interventions With Monthly Antibiotic Use, January 2007 to December 2018

Parameter	Piperacillin-tazobactam and carbapenems		Other broad-spectrum antibiotics	
	Estimate (95% CI), DDD per 1000 patient-days per mo	*P* value	Estimate (95% CI), DDD per 1000 patient-days per mo	*P* value
Intercept at time zero	52.19 (49.07 to 55.3)	<.001	1424.51 (1394.85 to 1454.18)	<.001
Preintervention trend	1.17 (0.99 to 1.35)	<.001	−0.05 (−1.92 to 1.83)	.96
Postintervention 1^a^				
Level change	−6.01 (−9.82 to −2.20)	<.002	103.46 (49.23 to 157.68)	<.001
Trend change	−0.84 (−1.06 to −0.62)	<.001	−11.05 (−15.55 to −6.55)	<.001
Trend	0.33 (0.18 to 0.48)	<.001	−11.1 (−15.12 to −7.08)	<.001
Postintervention 2^b^				
Level change	8.45 (2.82 to 14.08)	.004	−50.78 (−121.8 to 20.24)	.16
Trend change	−0.55 (−0.74 to −0.36)	<.001	9.00 (4.75 to 13.25)	<.001
Trend	−0.22 (−0.33 to −0.10)	.003	−2.10 (−3.13 to −1.07)	.001
Postintervention 3^c^				
Level change	8.29 (2.63 to 13.94)	.004	109.2 (57.79 to 160.61)	<.001
Trend change	0.50 (0.21 to 0.79)	.001	1.31 (−0.99 to 3.61)	.26
Trend	0.28 (0.02 to 0.55)	.04	−0.79 (−2.84 to 1.26)	.45

Abbreviation: DDD, defined daily dose.

^a^ Intervention 1 began in April 2009 and included introduction of empirical antibiotic guidelines and prospective review and feedback.

^b^ Intervention 2 began in April 2011 and included compulsory use of computerized decision support systems in addition to the voluntary access to the computerized decision support systems for antibiotic recommendations.

^c^ Intervention 3 began in March 2017 and included lifting of the compulsory use of computerized decision support systems for piperacillin-tazobactam and carbapenems for half of the wards in the hospital. Compulsory use was reinstated from September 2017.

In January 2007, the level of other broad-spectrum antibiotic use was 1424.51 (95% CI, 1394.85 to 1454.18) DDDs per 1000 patient-days, and the monthly use trend remained stable at −0.05 (95% CI, −1.92 to 1.83) DDDs per 1000 patient days. After intervention 1, there was a significant increase of 103.46 (95% CI, 49.23 to 157.68) more DDDs per 1000 patient-days in use level, followed by a significant decrease of −11.05 (95% CI, −15.55 to −6.55) fewer DDDs per 1000 patient-days per month in monthly trend compared with the preintervention trend. After intervention 2, there was a significant increase in monthly trend of 9.00 (95% CI, 4.75 to 13.25) more DDDs 1000 patient-days per month compared with the postintervention 1 trend, resulting in a lower rate of use at −2.10 (95% CI, −3.13 to −1.07) fewer DDDs per 1000 patient-days per month. After intervention 3, use level increased by 109.2 (95% CI, 57.79 to 160.61) DDDs per 1000 patient-days in the month of implementation and the monthly trend of other broad-spectrum antibiotics remained stable. (Table 1; Figure 1B) Antibiotic-specific results for interventions 1 through 3 are presented in eTable 1 in the Supplement.

### Incidence Density of MDROs

**Figure 2.**
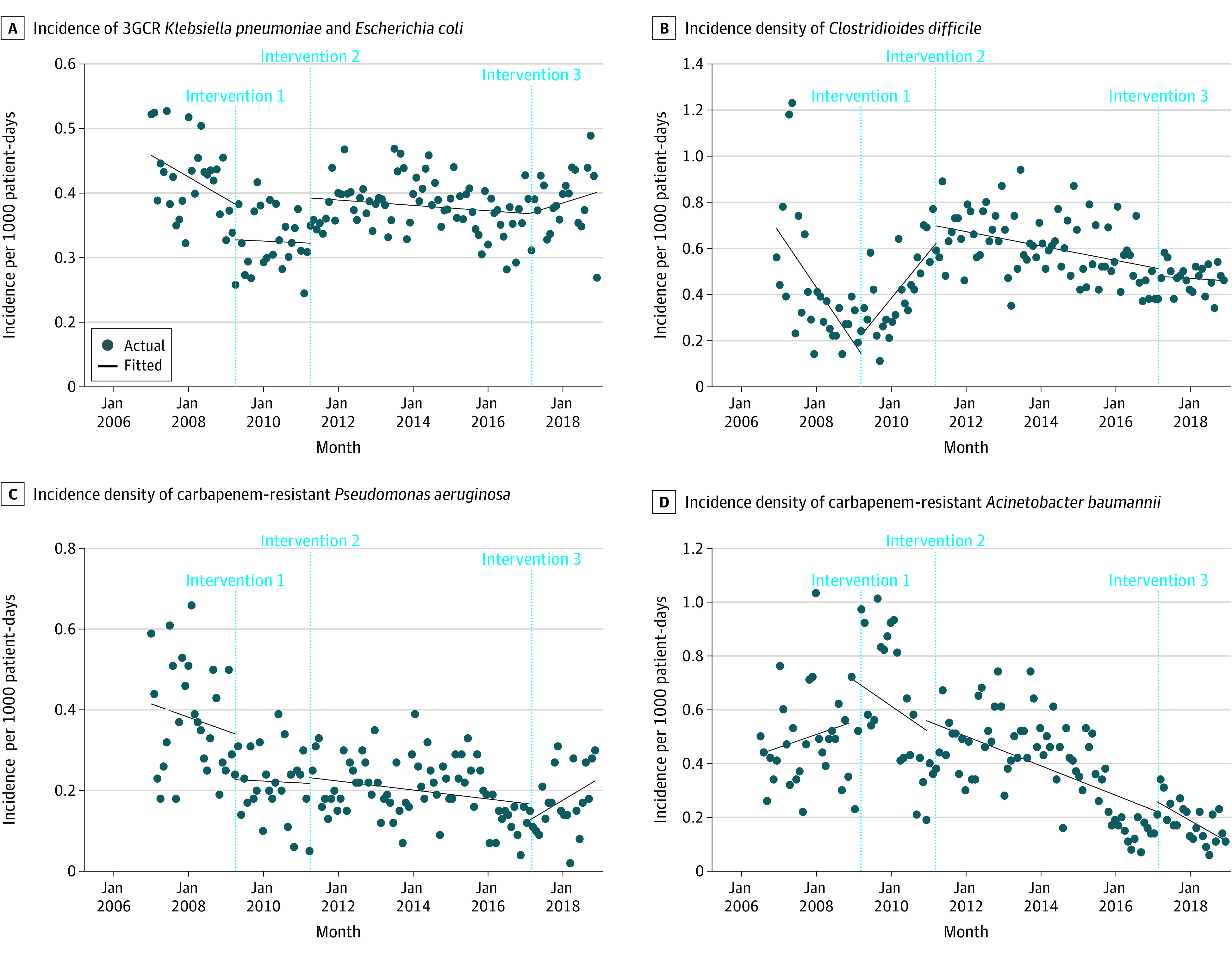
Incidence Density of Drug Resistant Organisms From 2007 to 2018 Intervention 1 began in April 2009 and included introduction of empirical antibiotic guidelines and prospective review and feedback. Intervention 2 began in April 2011 and included compulsory use of CDSS in addition to the voluntary access to the computerized decision support systems for antibiotic recommendations. Intervention 3 began in March 2017 and included lifting of the compulsory use of computerized decision support systems for piperacillin-tazobactam and carbapenems for half of the wards in the hospital. Compulsory use was reinstated from September 2017. 3GCR indicates third-generation cephalosporin–resistant.

There were no significant changes in the incidence density of 3GCR *K pneumoniae* and *E coli* after any of the interventions except for an increase in level in the month of implementation of intervention 2. (Figure 2A; Table 2) There was no significant correlation between the individual use of piperacillin-tazobactam, carbapenems, and other broad-spectrum antibiotics with the incidence density of 3GCR *K pneumoniae* and *E coli* (eTable 2 in the Supplement).

**Table 2.** Interrupted Time Series Analysis of the Association of Interventions With Monthly Antimicrobial Resistance Incidence, January 2007 to December 2018

Parameter	3GCR *K pneumoniae* and *E coli*		*C difficile*		Carbapenem-resistant *P aeruginosa*		Carbapenem-resistant *A baumannii*	
	Estimate (95% CI), per 1000 patient-days per mo	*P* value	Estimate (95% CI), per 1000 patient-days per mo	*P* value	Estimate (95% CI), per 1000 patient-days per mo	*P* value	Estimate (95% CI), per 1000 patient-days per mo	*P* value
Intercept at time zero	4.59 (4.14 to 5.04)	<.001	0.68 (0.46 to 0.91)	<.001	0.42 (0.30 to 0.53)	<.001	0.44 (0.33 to 0.54)	<.001
Preintervention trend	−0.03 (−0.05 to −0.002)	.04	−0.02 (−0.03 to −0.01)	.001	−0.003 (−0.01 to 0.005)	.43	0.004 (−0.002 to 0.01)	.21
Postintervention 1^a^								
Level change	−0.55 (−1.11 to 0.02)	.06	0.08 (−0.08 to 0.24)	.33	−0.11 (−0.22 to −0.01)	.03	0.17 (−0.10 to 0.45)	.21
Trend change	0.03 (−0.01 to 0.06)	.19	0.037 (0.02 to 0.05)	<.001	0.002 (−0.01 to 0.01)	.57	−0.01 (−0.03 to 0.01)	.20
Trend	−0.002 (−0.03 to 0.03)	.89	0.017 (0.010 to 0.024)	<.001	−0.0004 (−0.004 to 0.003)	.81	−0.01 (−0.03 to 0.01)	.35
Postintervention 2^b^								
Level change	0.70 (0.29 to 1.10)	.001	0.08 (−0.04 to 0.19)	.19	0.01 (−0.06 to 0.09)	.66	0.04 (−0.21 to 0.28)	.77
Trend change	−0.001 (−0.03 to 0.03)	.93	−0.02 (−0.03 to −0.01)	<.001	−0.0005 (−0.004 to 0.003)	.76	0.004 (−0.01 to 0.02)	.69
Trend	−0.004 (−0.01 to 0.001)	.14	−0.0026 (−0.004 to −0.0012)	<.001	−0.001 (−0.002 to 0.000)	.06	−0.005 (−0.01 to −0.003)	<.001
Postintervention 3^c^								
Level change	0.02 (−0.42 to 0.45)	.94	−0.03 (−0.12 to 0.05)	.42	−0.04 (−0.09 to 0.01)	.14	0.04 (−0.06 to 0.13)	.45
Trend change	0.02 (−0.03 to 0.06)	.40	0.0017 (−0.003 to 0.010)	.49	0.006 (0.002 to 0.01)	.007	−0.002 (−0.01 to 0.002)	.33
Trend	0.02 (−0.03 to 0.06)	.49	−0.0009 (−0.010 to 0.0038)	.71	0.005 (0.001 to 0.01)	.002	−0.007 (−0.01 to −0.003)	<.001

Abbreviations: 3GCR, third-generation cephalosporin–resistant; *A baumannii*, *Acinetobacter baumannii*; *C difficile*, *Clostridioides difficile*; *E coli*, *Escherichia coli*; *K pneumoniae*, *Klebsiella pneumoniae*; *P aeruginosa*, *Pseudomonas aeruginosa*.

^a^ Intervention 1 began in April 2009 and included introduction of empirical antibiotic guidelines and prospective review and feedback.

^b^ Intervention 2 began in April 2011 and included compulsory use of computerized decision support systems in addition to the voluntary access to the computerized decision support systems for antibiotic recommendations.

^c^ Intervention 3 began in March 2017 and included lifting of the compulsory use of computerized decision support systems for piperacillin-tazobactam and carbapenems for half of the wards in the hospital. Compulsory use was reinstated from September 2017.

In January 2007, the incidence density of *C difficile* was 0.68 cases per 1000 patient-days, with a decreasing trend of −0.02 (95% CI, −0.03 to −0.01) fewer cases per 1000 patient-days per month. The incidence density increased at a trend of 0.02 (95% CI, 0.01 to 0.02) more cases per 1000 patient-days per month after intervention 1, and it decreased at a trend of −0.0026 (95% CI, −0.004 to −0.0012) fewer cases per 1000 patient-days per month after intervention 2 (Figure 2B; Table 2). There were no significant changes after intervention 3. Use of carbapenems was most correlated with *C difficile* incidence density (*r* = 0.20; *P* = .02), with a lead time of 3 months (eTable 2 in the Supplement). Significant correlation was seen between other broad-spectrum antibiotic utilization and *C difficile* incidence density (*r* = 0.195 at 0-month lag; *P* = .02). Use of intravenous amoxicillin-clavulanate was also correlated with *C difficile* incidence density (*r* = 0.20 at 0-month lag; *P* = .02).

In January 2007, the incidence density of carbapenem-resistant *P aeruginosa* was 0.42 cases per 1000 patient-days. In the month of intervention 1, carbapenem-resistant *P aeruginosa* recorded a level reduction of 0.11 (95% CI, −0.22 to −0.01) fewer cases per 1000 patient-days. After intervention 3, there was a significant increase of 0.006 (95% CI, 0.002 to 0.010) more cases per 1000 patient-days per month in the monthly trend compared with the postintervention 2 trend (Figure 2C; Table 2) The incidence density correlated with use of piperacillin-tazobactam (*r* = −0.18 at 7-month lag; *P* = .03) and carbapenems (*r* = 0.22 at 1-month lag; *P* = .01) (eTable 2 in the Supplement).

In January 2007, the incidence density of carbapenem-resistant *A baumannii* was 0.44 cases per 1000 patient-days. The only significant change was observed after interventions 2 and 3. The incidence density decreased at a trend of 0.005 (95% CI, −0.01 to −0.003) fewer cases per 1000 patient-days per month after intervention 2 and 0.007 (95% CI −0.01 to, −0.003) fewer cases per 1000 patient-days per month after intervention 3 (Figure 2D; Table 2). No antibiotics were significantly correlated with carbapenem-resistant *A baumannii* incidence (eTable 2 in the Supplement).

### Unintended Outcomes

**Figure 3.**
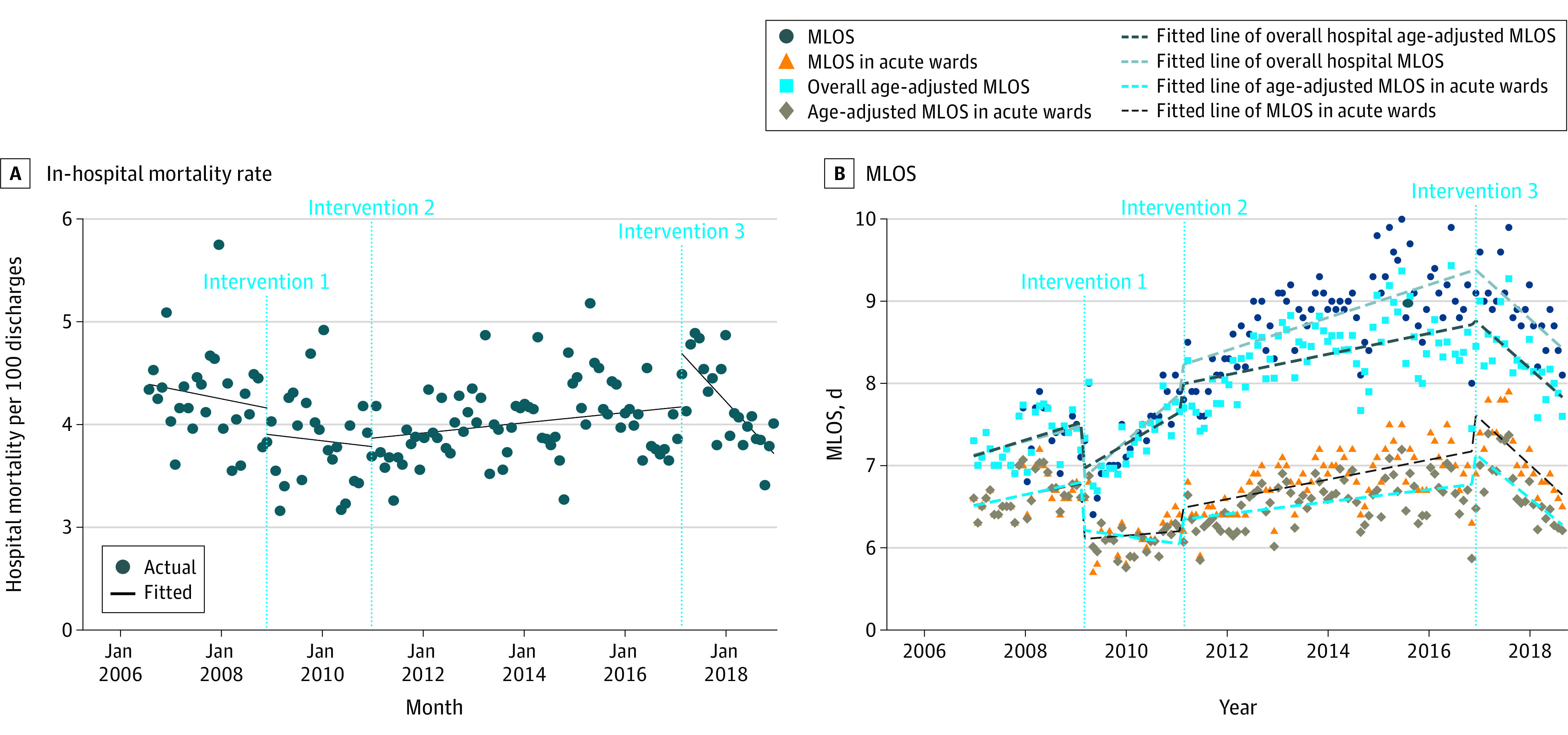
In-Hospital Mortality and Mean Length of Stay (MLOS) From 2007 to 2018 Intervention 1 began in April 2009 and included introduction of empirical antibiotic guidelines and prospective review and feedback. Intervention 2 began in April 2011 and included compulsory use of computerized decision support systems in addition to the voluntary access to the computerized decision support systems for antibiotic recommendations. Intervention 3 began in March 2017 and included lifting of the compulsory use of computerized decision support systems for piperacillin-tazobactam and carbapenems for half of the wards in the hospital. Compulsory use was reinstated from September 2017.

In-hospital mortality remained stable after interventions 1 and 2. After intervention 3, mortality decreased at a trend of −0.05 (95% CI, −0.08 to −0.03) fewer deaths per 100 discharges per month (Figure 3A). Age-adjusted LOS increased at a rate of 0.03 (95% CI, 0.004 to 0.05) more days per month in the month of intervention 1 and 0.01 (95% CI, 0.005, 0.016) more days per month in the month of intervention 2 (Figure 3B). After intervention 3, age-adjusted LOS decreased at a trend of −0.04 (95% CI, −0.06 to −0.03) fewer days per month. Similar trends were observed after excluding subacute care wards (eTable 3 and eTable 4 in the Supplement).

## Discussion

This cohort study found that implementation of PRF and CDSS was associated with limiting the use of piperacillin-tazobactam and carbapenems while reducing use of other broad-spectrum antibiotics in a hospital with high–antibiotic resistance rates. The continued use of CDSS was needed to sustain the decrease in use of piperacillin-tazobactam and carbapenem. There was a reduction in the incidence density of *C difficile* and carbapenem-resistant *P aeruginosa* after intervention 2. In-hospital mortality remained stable throughout the study, but there was an increase in age-adjusted LOS after interventions 1 and 2.

The reduction in piperacillin-tazobactam, carbapenems, and other broad-spectrum antibiotic use observed in our study was consistent with other successful antimicrobial stewardship program implementations.^16^ One of the concerns for implementation of antimicrobial stewardship programs is the undesirable consequence of the “squeezing the balloon” phenomenon,^17^ which is an increase in use of some antibiotics owing to restriction of the use of other antibiotics. Although we were not able to report total antibiotic use, we attempted to address this limitation by adding intravenous amoxicillin-clavulanate acid and other broad-spectrum antibiotics from the WHO Watch list.^1^

After compulsory CDSS use for piperacillin-tazobactam and carbapenems (intervention 3) was removed for half of the acute care wards, the monthly use of piperacillin-tazobactam and carbapenems increased while that of broad-spectrum antibiotics remained stable. Without compulsory CDSS, there could have been fewer opportunities for prescribers to consider alternate antibiotics and potentially an increase in use of piperacillin-tazobactam and carbapenems.^11,18^ The continued availability of the voluntary use of CDSS may have helped prescribers learn and adopt hospital empirical antibiotic guidelines, which were focused on antibiotic choice and duration, sustaining the trend of other broad-spectrum antibiotics.

The incidence density of 3GCR *K pneumoniae* and *E coli* remained stable throughout the study while that of *C difficile* decreased when compulsory CDSS was added. The decrease in *C difficile* also correlated with the decrease in other broad-spectrum antibiotic use. Carbapenem-resistant *P aeruginosa* incidence decreased after interventions 1 and 2 and increased after intervention 3, and changes correlated significantly with the use of carbapenems and piperacillin-tazobactam. Although the overall incidence of carbapenem-resistant *A baumannii* decreased throughout the years, it did not correlate with antibiotic use, which suggests that the decreasing trend could have been the result of infection control measures.^19^ Antimicrobial stewardship has been demonstrated to reduce the incidence of multidrug resistant and extended-spectrum β-lactamase (ESBL)–producing gram-negative bacteria in addition to incidence of *C difficile* infections.^20,21,22,23^ The presence of community acquired ESBL-producing gram negative bacteria, with reports ranging from 12% to 26% of all ESBL-producing gram-negative bacteremia being community acquired, may explain the stable trend of 3GCR *K pneumoniae* and *E.coli* in our study.^24,25^ A study by Bond et al^26^ demonstrated a similar decrease in trend of health care–associated *C difficile* infection after implementation of CDSS with concurrent guideline dissemination.

We evaluated our antimicrobial stewardship activities according to a structured framework of expected, desirable outcomes (intervention goals); expected, undesirable outcomes (intervention trade-offs); unexpected, undesirable outcomes (unpleasant surprises); and unexpected, desirable outcomes (pleasant surprises).^27^ In our study, there was an increase in age-adjusted LOS, which could be attributed to increases in proportions of patients older than 65 years and number of subacute care wards. Our previous study on the safety of PRF did not find a significant increase in LOS of patients who had carbapenem therapy deescalation.^28^ In addition, to address the challenges of an aging patient population, TTSH introduced its transitional care services in July 2016 to support timely transition from a hospital to the community, which may lead to reduction in age-adjusted LOS after intervention 3.^29^ A Cochrane review of antimicrobial stewardship interventions found consistent reduction in LOS in the included studies.^30^ No changes in in-hospital mortality rate were observed after antimicrobial stewardship interventions, which suggest that that our antimicrobial stewardship interventions were not associated with adverse outcomes in patients.

### Limitations

This study has several limitations. We did not include overall antibiotic use in the hospital and only focused on commonly used broad-spectrum antibiotics from the Watch list, based on WHO classification,^31^ as these were regarded as targets of stewardship programs and monitoring. We were unable to determine if the MDROs were community-acquired or health care–associated and did not account for the possible impact of infection control measures. There was no control group for comparative analysis, and confounding effects from other hospitalwide activities were not accounted for. The antibiotic use, resistance, and other outcomes may not have a direct association with PRF and CDSS, as prescribers may learn from interactions with our interventions and modified their overall antibiotic prescribing. We attempted to partially mitigate the possibility of chance finding by examining antibiotic use by individual antibiotics to check whether the trend was consistent across antibiotic groups.

## Conclusions

This cohort study found that implementation of PRF and CDSS was associated with limiting the use of piperacillin-tazobactam and carbapenems while reducing that of other antibiotics. The continued use of CDSS may be needed to sustain the reduction in piperacillin-tazobactam and carbapenems. Our antibiotic stewardship activities were associated with a reduction in *C difficile* and carbapenem-resistant *P aeruginosa* incidence without increasing mortality in a large university teaching hospital with high rates of antibiotic use and resistance over a 11-year period.

## Notes

Supplement. **eTable 1.** Interrupted Time Series Analysis of Monthly Antibiotic Utilization **eTable 2.** Cross-Correlation Function of MDROs and Antibiotic Utilizations **eTable 3.** Interrupted Time Series Analysis of Age-Adjusted Mean Length of Stay **eTable 4.** Interrupted Time Series Analysis of Monthly Patient Outcomes
