# Supplementary material for: Sustaining Antimicrobial Stewardship in a High–Antibiotic Resistance Setting
Source: JAMA Netw Open. 2022 May 3;5(5):e2210180. doi: 10.1001/jamanetworkopen.2022.10180 (PMC9066280; doi:10.1001/jamanetworkopen.2022.10180)
Supplement: Supplement. — eTable 1. Interrupted Time Series Analysis of Monthly Antibiotic Utilization eTable 2. Cross-Correlation Function of MDROs and Antibiotic Utilizations eTable 3. Interrupted Time Series Analysis of Age-Adjusted Mean Length of Stay eTable 4. Interrupted Time Series Analysis of Monthly Patient Outcomes [file jamanetwopen-e2210180-s001.pdf]

Supplemental Online Content

Ng TM, Heng ST, Chua BH, et al. Sustaining antimicrobial stewardship in a high–antibiotic resistance setting. *JAMA Netw Open*. 2022;5(5):e2210180. doi:10.1001/jamanetworkopen.2022.10180

eTable 1. Interrupted Time Series Analysis of Monthly Antibiotic Use

eTable 2. Cross-Correlation Function of MDROs and Antibiotic Use

eTable 3. Interrupted Time Series Analysis of Age-Adjusted Mean Length of Stay

eTable 4. Interrupted Time Series Analysis of Monthly Patient Outcomes

This supplemental material has been provided by the authors to give readers additional information about their work.

© 2022 Ng TM et al. *JAMA Network Open.*

eTable 1. Interrupted Time Series Analysis of Monthly Antibiotic Use

	**Piperacillin-tazobactam**			**Carbapenem**			**Co-amoxiclav (IV)**		
	**Est.**	**95% CI**	***P***	**Est.**	**95% CI**	***P***	**Est.**	**95% CI**	***P***
**Intercept at time zero**	22.93	21.26 to 24.6	<0.001	29.25	27.00 to 31.50	<0.001	827.36	807.26 to 847.45	<0.001
**Pre-intervention trend**	0.50	0.36 to 0.64	<0.001	0.67	0.52 to 0.82	<0.001	1.64	0.24 to 3.04	0.022
**Level change after intervention 1**	2.53	-0.99 to 6.05	0.158	-8.54	-11.23 to -5.84	<0.001	108.4	58.01 to 158.8	<0.001
**Trend change after intervention 1**	-0.25	-0.48 – 0.03	0.029	-0.59	-0.85 to -0.32	<0.001	-6.61	-10.16 to -3.07	<0.001
**Post intervention 1 trend**	0.25	0.07 to 0.43	0.008	0.08	-0.11 to 0.28	0.391	-4.97	-8.22 to -1.72	0.003
**Level change after intervention 2**	0.80	-2.14 to 3.74	0.591	7.65	1.73 to 13.57	0.012	-34.59	-84.56 to 15.38	0.173
**Trend change after intervention 2**	-0.36	-0.54 to -0.17	<0.001	-0.19	-0.41 to 0.03	0.091	4.31	1.01 to 7.6	0.011
**Post intervention 2 trend**	-0.11	-0.16 to -0.07	<0.001	-0.1	-0.2 to -0.01	0.034	-0.66	-1.17 to -0.16	0.0103
**Level change after intervention 3**	3.79	-0.21 to 7.79	0.063	4.5	-1.28 to 10.28	0.126	62.84	25.3 to 100.39	<0.001
**Trend change after intervention 3**	0.1	-0.16 to 0.36	0.463	0.4	0.07 to 0.74	0.018	-0.66	-3.52 to 0.87	0.564
**Post intervention 3 trend**	-0.02	-0.27 to 0.24	0.8993	0.3	-0.02 to 0.61	0.062	-1.32	-3.52 to 0.87	0.236

Interventions: (1) April 2009, Introduction of empiric antibiotic guidelines and PRF, (2) April 2011, Compulsory use of CDSS in addition to the voluntarily access of the CDSS for antibiotic recommendations and (3) March 2017, Compulsory use of CDSS for piperacillin-tazobactam and carbapenems were lifted for half of the wards in the hospital and re-instated from September 2017.
CDSS, computerized decision support systems; Est., estimate; CI, confidence interval; PRF, prospective review and feedback

© 2022 Ng TM et al. *JAMA Network Open.*

eTable 1 cont'd

	Fluoroquinolones			Third generation cephalosporins			Vancomycin		
	**Est.**	**95% CI**	***P***	**Est.**	**95% CI**	***P***	**Est.**	**95% CI**	***P***
**Intercept at time zero**	454.88	445.79 to 463.97	<0.001	120.5	116.38 to 124.61	<0.001	20.93	17.79 to 24.08	<0.001
**Pre-intervention trend**	-1.09	-1.86 to -0.32	0.006	-0.92	-1.35 to -0.49	<0.001	0.32	0.10 to 0.53	0.004
**Level change after intervention 1**	-14.34	-35.55 to 6.87	0.184	5.73	-7.58 to 19.04	0.396	-0.56	-4.27 to 3.16	0.767
**Trend change after intervention 1**	-2.77	-3.99 to -1.55	<0.001	-1.34	-2.1 to -0.59	0.001	-0.46	-0.7 to -0.23	<0.001
**Post intervention 1 trend**	-3.86	-4.85 to -2.86	<0.001	-2.26	-2.9 to -1.62	<0.001	-0.14	-0.25 to -0.04	0.006
**Level change after intervention 2**	-29.07	-50.7 to -7.43	<0.001	5.23	-2.71 to 13.17	0.195	6.49	3.27 to 9.71	<0.001
**Trend change after intervention 2**	2.31	1.18 to 3.44	<0.001	2.24	1.59 to 2.89	<0.001	0.15	0.03 to 0.26	0.014
**Post intervention 2 trend**	-1.54	-1.98 to -1.11	<0.001	-0.02	-0.12 to 0.07	0.644	0	-0.05 to 0.06	0.934
**Level change after intervention 3**	59.9	36.0 0 to 83.80	<0.001	-7.96	-12.74 to -3.17	0.001	-3.22	-5.7 to -0.73	0.012
**Trend change after intervention 3**	1.55	0.54 to 2.55	0.003	0.18	-0.26 to 0.61	0.42	0.28	0.13 to 0.43	<0.001
**Post intervention 3 trend**	0	-0.99 to 1	0.995	0.15	-0.27 to 0.58	0.472	0.28	0.15 to 0.42	0.146

Interventions: (1) April 2009, Introduction of empiric antibiotic guidelines and PRF, (2) April 2011, Compulsory use of CDSS in addition to the voluntarily access of the CDSS for antibiotic recommendations and (3) March 2017, Compulsory use of CDSS for piperacillin-tazobactam and carbapenems were lifted for half of the wards in the hospital and re-instated from September 2017.
CDSS, computerized decision support systems; Est., estimate; CI, confidence interval; PRF, prospective review and feedback

© 2022 Ng TM et al. *JAMA Network Open.*

eTable 2. Cross-Correlation Function of MDROs and Antibiotic Use

Antibiotic group	Peak correlation (r)	Lag (month)	*P*-value
**Piperacillin-tazobactam**	-0.160	9	0.055
**Carbapenems**	-0.153	8	0.066
**Other broad-spectrum antibiotics**	0.078	10	0.349
**Co-amoxiclav (IV)**	0.148	10	0.076
**Fluoroquinolones**	0.136	0	0.104
**Third- generation cephalosporins**	0.216	1	0.010

eTable 2 cont'd

Antibiotic group	Peak correlation (r)	Lag (month)	*P*-value
**Piperacillin-tazobactam**	-0.104	5	0.214
**Carbapenems**	0.196	3	0.018
**Oher broad-spectrum antibiotics**	0.195	0	0.019
**Co-amoxiclav (IV)**	0.197	0	0.018
**Fluoroquinolones**	0.117	10	0.162
**Third generation cephalosporins**	0.111	0	0.181

eTable 2 cont'd

Antibiotic group	Peak correlation (r)	Lag (month)	*P*-value
**Piperacillin-tazobactam**	-0.182	7	0.029
**Carbapenems**	0.216	1	0.010
**Oher broad-spectrum antibiotics**	0.147	4	0.078
**Co-amoxiclav (IV)**	0.145	4	0.082
**Fluoroquinolones**	0.030	1	0.718

© 2022 Ng TM et al. *JAMA Network Open.*

**Third generation cephalosporins**	0.052	12	0.530

eTable 2 cont'd

Antibiotic group	Peak correlation (r)	Lag (month)	*P*-value
**Piperacillin-tazobactam**	0.127	1	0.127
**Carbapenems**	-0.127	3	0.128
**Oher broad-spectrum antibiotics**	0.100	10	0.227
**Co-amoxiclav (IV)**	0.208	2	0.012
**Fluoroquinolones**	-0.142	4	0.088
**Third generation cephalosporins**	0.127	11	0.128

© 2022 Ng TM et al. *JAMA Network Open.*

eTable 3. Interrupted Time Series Analysis of Age-Adjusted Mean Length of Stay

	**All wards**			**Acute wards excluding subacute wards**		
	**Est.**	**95% CI**	***P***	**Est.**	**95% CI**	***P***
**Intercept at time zero**	7.11	6.96 to 7.26	<0.001	6.51	6.38 to 6.63	<0.001
**Pre-intervention trend**	0.02	0.01 to 0.03	0.003	0.01	0.00 to 0.02	0.008
**Level change after intervention 1**	-0.57	-1.02 to -0.11	0.015	-0.6	-0.95 to -0.24	0.001
**Trend change after intervention 1**	0.01	-0.01 to 0.04	0.302	-0.02	-0.04 to 0	0.096
**Post intervention 1 trend**	0.03	0.00 to 0.05	0.023	-0.01	-0.03 to 0.01	0.492
**Level change after intervention 2**	0.32	0.00 to 0.63	0.047	0.31	0.05 to 0.57	0.022
**Trend change after intervention 2**	-0.02	-0.05 to 0.01	0.145	0.01	-0.01 to 0.03	0.209
**Post intervention 2 trend**	0.01	0.00 to 0.02	<0.001	0.01	0.02 to 0.7	0.038
**Level change after intervention 3**	0.04	-0.28 to 0.36	0.824	0.36	0.02 to 0.7	0.038
**Trend change after intervention 3**	-0.05	-0.07 to -0.04	<0.001	-0.05	-0.07 to -0.02	<0.001
**Post intervention 3 trend**	-0.04	-0.06 to -0.03	<0.001	-0.04	-0.06 to -0.02	<0.001

Interventions: (1) April 2009, Introduction of empiric antibiotic guidelines and PRF, (2) April 2011, Compulsory use of CDSS in addition to the voluntarily access of the CDSS for antibiotic recommendations and (3) March 2017, Compulsory use of CDSS for piperacillin-tazobactam and carbapenems were lifted for half of the wards in the hospital and re-instated from September 2017.
CDSS, computerized decision support systems; Est., estimate; CI, confidence interval; PRF, prospective review and feedback

© 2022 Ng TM et al. *JAMA Network Open.*

eTable 4. Interrupted Time Series Analysis of Monthly Patient Outcomes

Parameter	Hospital mortality (deaths/100 discharges)			Age-adjusted average length of stay (days)		
	Est.	95% CI	*P*-value	Est.	95% CI	*P*-value
**Intercept at time zero**	4.39	4.14 to 4.65	<0.001	7.11	6.96 to 7.26	<0.001
**Pre-intervention trend**	-0.01	--0.03 to 0.01	0.322	0.02	0.01 to 0.03	0.003
**Level change after intervention 1**	-0.26	-0.75 to 0.23	0.301	-0.57	-1.02 to -0.11	0.015
**Trend change after intervention 1**	0.00	-0.03 to 0.03	0.815	0.01	-0.01 to 0.04	0.302
**Post intervention 1 trend**	-0.005	-0.03 to 0.02	0.693	0.03	0.00 to 0.05	0.023
**Level change after intervention 2**	0.08	-0.3 to 0.47	0.675	0.32	0.00 to 0.63	0.047
**Trend change after intervention 2**	0.01	-0.02 to 0.03	0.468	-0.02	-0.05 to 0.01	0.145
**Post intervention 2 trend**	0.004	0.00 to 0.01	0.052	0.01	0.005 to 0.02	<0.01
**Level change after intervention 3**	0.52	0.17 to 0.86	0.004	0.04	-0.28 to 0.36	0.824
**Trend change after intervention 3**	-0.05	-0.07 to -0.03	<0.001	-0.05	-0.07 to -0.04	<0.01
**Post intervention 3 trend**	-0.05	-0.07 to -0.03	<0.001	-0.04	-0.06 to -0.03	<0.01

Interventions: (1) April 2009, Introduction of empiric antibiotic guidelines and PRF, (2) April 2011, Compulsory use of CDSS in addition to the voluntarily access of the CDSS for antibiotic recommendations and (3) March 2017, Compulsory use of CDSS for piperacillin-tazobactam and carbapenems were lifted for half of the wards in the hospital and re-instated from September 2017.

CDSS, computerized decision support systems; Est., estimate; CI, confidence interval; PRF, prospective review and feedback

© 2022 Ng TM et al. *JAMA Network Open.*
